# Decoding Stress Responses in Farmed Crustaceans: Comparative Insights for Sustainable Aquaculture Management

**DOI:** 10.3390/biology14080920

**Published:** 2025-07-23

**Authors:** Fitriska Hapsari, Muhammad Agus Suprayudi, Dean M. Akiyama, Julie Ekasari, Parisa Norouzitallab, Kartik Baruah

**Affiliations:** 1 Department of Aquaculture, Faculty of Fisheries and Marine Sciences, IPB University, Bogor 16680, Indonesia; ikasutrisno@apps.ipb.ac.id (F.H.); muhammadsu@apps.ipb.ac.id (M.A.S.); 2 Department of Technical Aquaculture, Jakarta Technical University of Fisheries, Jakarta 12520, Indonesia; 3 PT. Central Proteina Prima, Jakarta 12190, Indonesia; akiyamadm@cpp.co.id; 4 Department of Applied Animal Science and Welfare, Faculty of Veterinary, Medicine and Animal Sciences, Swedish University of Agricultural Sciences, 75007 Uppsala, Sweden; parisa.norouzitallab@slu.se

**Keywords:** aquaculture, crustacean, stress mitigation, stressors, stress response, stress indicator

## Abstract

Aquaculture is a crucial food-producing sector that can supply more essential nutrients to nourish the growing human population. However, it faces challenges, including limited water quality and space competition. These constraints have led to the intensification of culture systems for more efficient resource use while maintaining or increasing production levels. However, intensification introduces stress risks to cultured organisms by, for instance, overcrowding, waste accumulation, and water quality deterioration, which can negatively affect the growth, health, and immunity of animals and cause diseases. Additionally, environmental changes due to climate and anthropogenic activities further intensify the environmental stress for aquaculture organisms, including crustaceans. Shrimp are one of the most widely cultured and consumed farmed crustacea. Relative to aquatic vertebrates such as fish, the physiology of crustaceans has simpler physiological structures, as they lack a spinal cord. Consequently, their stress response mechanisms follow a single pathway, resulting in less complex responses to stress exposure compared to those of fish. While stress is considered a primary factor influencing the growth, health, and immunity of shrimp, comprehensive research on crustacean stress responses remains limited. Understanding the stress response at the organismal and cellular levels is essential to identify sensitive and effective stress biomarkers which can inform the development of targeted intervention strategies to mitigate stress. This review provides a comprehensive overview of the physiological changes that occur in crustaceans under stress, including hormonal, metabolic, hematological, hydromineral, and phenotypic alterations. By synthesizing current knowledge, this article aims to bridge existing gaps and provide insights into the stress response mechanisms, paving the way for advancements in crustacean health management.

## Simple Summary

Aquaculture is vital for providing essential nutrients to the growing human population, but it faces challenges such as limited water quality and space. To address these, aquaculture systems have become more intensive, but this also introduces stress risks to cultured organisms, such as overcrowding, waste buildup, and water quality deterioration, which can harm their growth, health, and immunity. Climate change and human activities further increase environmental stress, especially for crustaceans like shrimp. Shrimp have a simpler physiology compared to fish, leading to less complex stress responses. Although stress is known to impact shrimp growth, health, and immunity, research on their stress responses is limited. Understanding these responses at both the organismal and cellular levels is crucial for identifying effective biomarkers and developing targeted strategies to reduce stress. This review summarizes the physiological changes in crustaceans under stress and aims to improve crustacean health management by filling knowledge gaps in stress response mechanisms.

## 1. Introduction

Driven by the increasing demand for aquatic products, the aquaculture sector has expanded rapidly over the past few decades. Over the past six decades, the share of feed consumption attributable to aquaculture has risen significantly, from just 6% in the 1960s to 56% in 2021 [1]. In 2020, aquaculture accounted for 56% of the aquatic products consumed by humans [2]. Shellfish consumption has also shown a notable increase over the same period, rising from 14% to 26%. Within this group, crustaceans, including shrimp, lobster, and crab, have demonstrated strong growth in both consumption and production. Crustaceans are the second most cultured aquatic organisms after finfish, and their production has increased from about 5.5 million tons in 2016 to about 7.5 million tons in 2022 (36% increase) [1]. While many crustacean species are cultivated for human consumption, others, such as copepods, play an important indirect role in aquaculture as live feed for the larval stages of fish and shrimp, thus supporting the production of higher-trophic-level species. Over the last two decades, shrimp production in China has seen the greatest increase, rising by 194% (from 1.11 million MT to 3.27 million MT), followed by Ecuador (12.69%), Argentina (3.33%), and Vietnam (3.14%). Shrimp production in India and Indonesia has also tripled during the same period. This growth is largely attributed to the intensification of shrimp aquaculture systems in these regions [3]. A meta-analysis examining the relationship between shrimp production intensity and land use in these countries revealed that Vietnam has the highest production intensity (36.66 t/ha/year), followed by Indonesia (22.85 t/ha/year) [4]. Despite the potential benefits, production intensification faces some major challenges, such as environmental changes and disease outbreaks.

Climate change imposes additional environmental pressures on aquaculture, including extreme weather events, temperature fluctuations, changes in sea surface salinity, shifting rainfall patterns, rising sea levels, and increased anthropogenic pollution. These factors have direct and indirect adverse effects on aquaculture production [2,5]. Although aquaculture is generally practiced in controlled environments, many operations, particularly pond-based and coastal systems, remain exposed to climatic variability. For instance, excessive rainfall or prolonged drought can alter pond salinity and temperature beyond optimal thresholds, disrupting water quality and animal health. These climate-driven stressors can compromise the effectiveness of control measures, especially in open or semi-enclosed systems such as earthen shrimp ponds. Multiple studies have demonstrated the detrimental consequences of climate change on the aquaculture industry, including total stock loss, increased mortality, decreased productivity, pond/tank damage, increased operational expenses, and reduced profitability [6,7,8], as well as a negative impact on the seafood supply chain [9]. The increase in global population significantly exacerbates the anthropogenic environmental footprint, such as pollution and eutrophication, resulting in the deterioration of water sources available for aquaculture activities. This further induces physiological responses such as metabolic stress and toxicity responses that lead to growth retardation, reduced immunity, or even mortality.

In an aquaculture system, disease outbreaks are closely linked to environmental quality, the animals’ physiological condition, the presence of pathogenic organisms, and their interrelations. When situated in a suboptimum environmental condition, most aquatic organisms will try to adapt and develop a series of metabolic responses in response to stress to overcome the changes and immediately return to homeostatic conditions. These processes will alter the overall bioenergetics in the animal’s body, where energy will mainly be allocated for metabolic adjustment, thus reducing the energy available for other physiological functions, such as the defense system and growth. Previous studies have demonstrated the adverse impacts of environmental changes, such as oxygen levels [10,11,12], temperature [13,14,15,16,17,18], salinity [19,20,21,22,23], pH [24,25,26], and ammonia concentrations [27,28,29,30,31], on crustacean species, especially shrimp. In those studies, environmental stressors were shown to cause a series of physiological dysfunctions, including a weakening of immunity, resulting in an increased susceptibility of the organisms to diseases. Accumulating evidence [11,12,16,32] suggests that stress strongly corresponds to disease susceptibility by both compromising the immunity of organisms and increasing the virulence factors of pathogens. Therefore, stress mitigation is crucial for improving the resilience of farmed crustaceans, particularly in the face of environmental challenges and increasing disease threats. To achieve effective stress mitigation strategies, a better understanding of stress responses in crustaceans is essential. However, only limited information in this area is available, highlighting an urgent need for comprehensive research to bridge this critical gap.

This literature review focuses on providing a thorough overview of stress response in crustaceans, highlighting stress biomarkers and potential strategies for mitigation. The structure of the review includes an overview of stress in aquaculture systems, covering the types and sources of stress, general stress responses in aquatic organisms, the physiology of stress in crustaceans starting from the neuroendocrine system to the immune response, differences in stress responses between crustaceans and fish (vertebrates), stress indicators, and mitigation strategies, as well as identifying gaps and future research directions.

## 2. Scope of Stress in Aquaculture

### 2.1. Definition of Stress

The concept of “stress” has been well defined in previous studies [33,34,35,36,37]. Stress is an essential mechanism in the physiological adaptation to stressors; it includes molecular and biochemical modifications in restoring homeostasis or behavioral responses such as escaping [38]. Despite the lack of a universally accepted definition, it is reasonable to define stress as a state generated by excessive exogenous stimuli (stressors) that induces adaptive responses of altered neuroendocrine, physiological, and behavioral states in an organism.

The stress level of an organism can be classified into two categories: eustress, which represents a low level of stress, and distress, which signifies a higher level of stress [34]. Eustress can be advantageous and can improve performance and have a favorable effect. Conversely, distress or chronic stress may result in both beneficial and detrimental behaviors, decreasing performance and threatening the well-being and overall health of the animal [34,36,39,40]. Dealing with long-term stress requires significant energy and resources [40], which suggests that the body’s response to stress is influenced by the type and intensity of the stressor [38].

**Figure 1:**
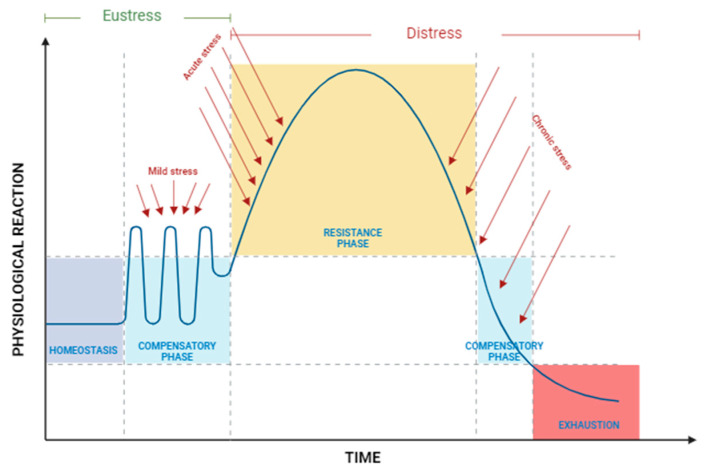
The relationship between physiological reactions and different types of stress and their durations.

Stressful conditions in cultured organisms typically arise from multiple interacting stressors. Various stressors can hinder growth, weaken immunity, diminish disease resistance, and possibly lead to mortality [35], which is related to the energy required to restore the organism to the equilibrium state. Therefore, the ability of an organism to survive a stressor will depend on its capability to return to initial homeostatic condition [34]. Figure 1 illustrates the physiological reactions that occur in the body when exposed to multiple stressors. Homeostasis is a state in which the body’s metabolic condition remains stable and balanced. Because of this function, energy homeostasis can be used as a tool to assess how tolerant an organism is to stress in the environment [41]. In response to mild stress, such as minor fluctuations in an unstable aquatic environment, the body’s metabolism may shift slightly. However, its regulatory mechanisms generally manage these conditions within an adaptive range, entering what is known as the compensatory phase. This form of mild stress, often classified as eustress, allows the organism to adapt without significant disruption (Figure 1).

When the intensity of the stressor increases, acute stress arises, resulting in pronounced physiological imbalances, including hormonal shifts and metabolic alterations [40,42]. This level of stress, along with chronic stress, is categorized as distress, as it exceeds the body’s adaptive capacity and can lead to adverse effects. In the acute stress state, the body enters the resistance phase, where it attempts to re-establish equilibrium or homeostasis [34]. However, with prolonged or chronic exposure to stressors, the adaptive mechanisms of the organism become exhausted, ultimately reaching an exhaustion phase that may lead to decline or even death [33,42,43].

### 2.2. Types and Sources of Stress

Stressors can be categorized based on the exposure duration and the source. Accordingly, stress response will also depend on the characteristics and intensity of the stressor as well as the duration of exposure, ranging from rapid recovery to severe imbalances and death [40,42].

#### 2.2.1. Stressors According to Exposure Duration and Intensity

Stress can manifest as either acute or chronic, with chronic stress, such as prolonged exposure to anthropogenic noise, potentially leading to significant physiological disruptions in fish (e.g., elevated cortisol levels, increased heart rate, and accelerated yolk sac depletion) and behavioral alterations (e.g., anxiety-like responses and impaired exploratory behavior) [34,43,44,45]. Most stressors in nature are considered acute because they occur due to demanding situations like predation or fighting. These stressors typically occur over a short period with a high level of intensity. If the outcome is successful, it can lead to a valuable learning experience for future situations [43]. Chronic situations, in contrast, occur when the intensity of the stressor is consistently low but continuous [40]. These typically involves extended durations [42,43] and a shift in energetic metabolism to fulfill the requirements of the stressors [36,40]. Prolonged and intense stress exposure can cause the stress response to lose its ability to adapt effectively, leading to growth suppression, reproductive failure, and reduced susceptibility to infections [33,42,43].

In farmed crustaceans, several external stressors can have acute effects. For instance, temperature changes in *Macrobranchium rosenbergii* and hypoxia in Pacific white shrimp *Litopenaeus vannamei* can lead to an increase in norepinephrine levels 30 and 120 min after exposure to stress, respectively [10,15]. In contrast, stressors such as ammonia-N exposure and salinity changes in *L. vannamei* result in a delayed, chronic response, with elevated norepinephrine levels observed after 6 and 12 h, respectively [20,21]. These findings suggest that the classification of a stressor as acute or chronic should be based on its physiological effects over time, not merely on the duration or intensity of the stressor itself.

#### 2.2.2. Stressors According to Source

In the context of an aquaculture system, stress can also be classified based on its source (Table 1). Environmental factors are the most common stressors in aquaculture [35,38,40,46] that may lead to physiological dysfunction in the animal [40,46,47]. These stressors can be categorized into physical, chemical, and biological stressors, while non-environmental stressors are considered procedural stressors (Table 1). Interestingly, although the physiological response and biochemistry processes pathways upon exposure may generally be similar, different stressors can result in different and specific responses and therefore different mitigation strategies.

Physical stressors, such as temperature, light, and noise, are considered the most common physical stressors that could disrupt the physiological and biochemistry processes of aquatic organisms, especially through oxidative stress pathways. Temperature stress, whether cold or heat, disrupts metabolism and energy allocation (ATP) in crustaceans. For example, prolonged cold exposure (10 °C for 72 h) in Kuruma shrimp (*Marsupenaeus japonicus*) [13] can result in cellular damage, while heat stress (an increase of 3 °C to 7 °C) in copepods triggers oxidative stress and eventually affects the egg production rate [48,49]. Photoperiods, light intensity, and light spectrum have varying effects across crustacean species and seems to be related to the circadian rhythm, which is regulated by melatonin, the light perception hormone [50]. Continuous light exposure could induce oxidative stress and immune suppression in crabs [51,52]. In addition to oxidative stress, prolonged darkness could also induce photoreceptor damage, which eventually suppresses animals’ visual sensitivity [53]. A light intensity that is too low or too high can not only lead to oxidative damage but also to the suppression of molting behavior through the downregulation of ecdysteroid hormone synthesis and the upregulation of mol-inhibition hormone (MIH) in *Scylla paramamosain* [54] and *Portunus trituberculatus* [55]. Interestingly, light spectrum can also trigger a similar stress response in *S. paramamosain,* as shown by Chen et al. [54], who reported that violet light increased cortisol levels and the upregulation of MIH. Noise pollution is another environmental factor capable of inducing stress responses in organisms. For example, studies on *L. vannamei* revealed that noise pollution led to elevated stress hormone levels. However, the increase was minimal, as no significant differences were observed compared to control groups maintained under normal conditions [56,57]. Furthermore, turbidity levels above 30 NTU in both *L. vannamei* and delta smelt (*Hypomesus transpacificus*) increased stress markers, compromised immune function, and increased mortality rates [58].

Chemical stressors, including dissolved oxygen (DO), ammonia, nitrite, nitrate, hardness, CO_2_, pH, alkalinity, salinity, feed, pollutants, xenobiotics, and toxins, significantly affect the physiological condition of most aquatic species. Under hypoxic conditions, both invertebrates and vertebrates exhibit similar adaptive responses aimed at conserving energy, marked by changes in Hypoxia Inducible Factor 1-α (HIF-1α) gene expression, along with carbohydrate and lipid metabolism [10,11,59].

In *L. vannamei*, the differences in biogenic amine responses in the hemolymph and eyestalk suggest that biogenic amines play a key role in mediating the body’s stress response and energy mobilization [10]. Exposure to ammonia, nitrite, and nitrate stress increased oxidative stress-related enzymes like catalase (CAT), malondialdehyde (MDA), superoxide dismutase (SOD), and glutathione (GSH), impacting growth and immunity [29,60,61,62,63,64]. Among these stressors, *L. vannamei* exhibited a stronger stress response to nitrite than ammonia [64]. Additionally, factors like water hardness and pH also contribute to stress in aquatic species. In contrast, pH stress reveals species-specific responses: shrimp show lower levels of glutathione s-transferase (GST), SOD, glutathione peroxidase (GPx), and MDA compared to controls [24].

Alkalinity stress leads to gill damage and deformation, along with the downregulation of genes associated with ion transport after prolonged exposure to alkalinity (48 h) in shrimp [65]. In *L. vannamei*, high CO_2_ concentrations (>88.0 mg/L) result in sharp mortality rates within the first 24 h [66]. Although shrimp are euryhaline, low salinity (3 g/L) triggers stress responses in the hepatopancreas, indicated by the differential expression of proteins involved in energy metabolism, detoxification, and lipid and protein metabolism [67]. Similarly, the freshwater prawn *M. nipponense* exhibits comparable responses under low-salinity conditions [68]. To cope with such stress, *L. vannamei* adjust the osmotic concentration of their hemolymph [69]. Other crustaceans, such as copepods, also experience alterations in isosmotic intracellular regulation (IIR) when exposed to both low- and high-salinity changes [70].

Exposure to heavy metal pollutants damages the gills, kidneys, and liver of crustaceans. *L. vannamei* exposed to cadmium and lead demonstrated significant elevations in oxidative stress markers such as SOD, MDA, and GSH activities [32]. Toxins from bacterial or fungal species can also induce lethal stress, as shown by differential gene expression analyses, which revealed alterations in antioxidant activity, lipid and carbohydrate metabolism, and protein synthesis in affected organisms [71], as well as disruptions in the molting process in copepods [72]. Dietary nutrient imbalance also induces stress. For instance, *L. vannamei* fed with varying carbohydrate/protein, lipid/protein, and energy/protein ratios exhibited altered growth performance and increased SOD activity in the digestive gland [73].

Biological factors, including parasitic pathogens, predator presence, and overcrowding, serve as significant stressors for aquatic organisms. Pathogen attacks—both viral and bacterial—on shrimp have been linked to heightened oxidative stress, marked by increased peroxidase enzymes in *Penaeus monodon* [74]. In oriental river prawns (*Macrobrachium nipponense*) exposed to the parasite *Tachaea chinensis*, oxidative stress indicators such as MDA, CAT, GST, and alkaline phosphatase (AKP) were notably elevated [75]. Predator threats may also be another biological stressor. For example, the presence of predators decreased the routine metabolic rate (RMR) as an adaptive mechanism to evade predation in spiny lobster due to their tendency to hide when predators are nearby [76]. Overcrowding is a biological stressor known to induce oxidative stress across aquatic species. A density of more than 45 PL/m^2^ in *L. vannamei* led to elevated SOD, CAT, GPx, blood glucose levels, and stress gene expression [77,78].

Procedural stressors in aquaculture systems include handling, confinement, disease treatment, feeding, and transportation. Handling is one of the most immediate stressors, rapidly elevating stress hormone levels. In shrimp, stress indicators appear in metabolic responses, such as fluctuations in hemolymph glucose, lactate, total lipid, and total protein levels [79]. A tenfold surge in dopamine is observed within the first 30 min following acute handling stress, along with increased glucose and lactate levels in the hemolymph of *L. vannamei* [80]. Additionally, nauplii of the copepod *Acartia tonsa* exhibit a higher survival rate than adults when subjected to handling [81]. Confinement of *L. vannamei* influences blood glucose levels and magnesium ions in the blood [82].

Transportation is one of the most common procedural stressors. Numerous studies have been conducted to identify the most efficient and low-stress transportation methods. One such method is water-free transport. A previous study [83] showed that the water-free transport of shrimp led to increased antioxidant enzyme activity in the hemolymph and muscle tissues. Conversely, a 100% survival rate was recorded for turbot fish transported without water for 24 h [84]. The key difference between these two studies lies in the temperature used during transport: *L. vannamei* shrimp were subjected to a temperature shock of 13 °C, whereas turbot fish were exposed to 2 °C. The higher temperature prevented the shrimp from entering a fully dormant state, leading to heightened stress during transportation.

**Table 1:** Common stressors encountered in crustacean aquaculture practice.

Type of Stressor	Stressors	Range	Species	Reference
Physical	Temperature	10 ± 0.5 °C	*Marsupenaeus japonicus*	[13]
	Light intensity and photoperiod	12 L:12 D–24 L:0 D	*Eriocheir sinensis*	[52]
	Sound	128 dB re 1 µPa	*Litopenaeus vannamei*	[57]
	Turbidity	30–120 NTU	*Litopenaeus vannamei*	[58]
Chemical	Dissolved oxygen	0.8–3.5 mg/L	*Litopenaeus vannamei*	[11]
	Ammonia	60.21 mg/L	*Litopenaeus vannamei*	[29]
	Nitrite	5–15 mg/L	*Scylla paramamosain*	[61]
	Nitrate	35–910 ppm	*Litopenaeus vannamei*	[63]
	Hardness	25–1000 mg/L	*Macrobrachium rosenbergii*	[85]
	pH	9.5	*Litopenaeus vannamei*	[24]
	Alkalinity	10 mmol/L	*Exopalaemon carinicauda*	[65]
	Dissolved CO	14.5–175.0 mg/L	*Litopenaeus vannamei*	[66]
	Salinity	3 ppt	*Litopenaeus vannamei*	[67]
	Feed	CBH:P 0.6–2.1; L:P 0.2–0.36	*Litopenaeus vannamei*	[73]
	Pollutants	500 μg/L Cd and 500 μg/L Pb	*Litopenaeus vannamei*	[32]
	Toxins	0 and 4 mg/kg	*Eriocheir sinensis*	[71]
Biological	Pathogens	100 µL of a 1 × 10^11^ CFU/L	*Penaeus monodon*	[86]
	Parasites	Not stated	*Macrobrachium nipponense*	[75]
	Predators	Open-flow kairomones	*Jasus edwardsii*	[76]
	Overcrowding	30–60 PL8/m^2^	*Litopenaeus vannamei*	[78]
Procedural	Handling and hauling	Every morning for 4 weeks	*Litopenaeus vannamei*	[79]
	Confinement	Bottom pond for 48 h	*Litopenaeus stylirostris*	[82]
	Disease treatment	1 mg/L for 1 week	*Litopenaeus vannamei*	[87]
	Feeding	1–5 times/day	*Procambarus clarkia*	[88]
	Transportation	Water-free for 10 h	*Litopenaeus vannamei*	[83]

## 3. Stress Response

In response to stress, an organism employs an adaptive mechanism to maintain homeostasis [33,35,36,37,40]. Chronic stress, however, impairs these adaptive mechanisms and negatively affects the immune system, growth, and reproduction [33]. The extent of response varies based on the type and duration of the stressor, the species, the stage of life, nutritional status, and overall physiological condition [34,35,36,89]. This is because the stress response involves multiple physiological mechanisms, including neuroendocrine regulation, gene and protein changes, energy metabolism, immune function, and metabolic adjustments [35,40].

The general adaptive syndrome (GAS) was identified as an organism’s adaptive physiological response to a stressor. It comprises three stages: first, an alarm reaction that triggers the secretion of stress hormones, such as catecholamines and corticosteroids; second, a resistance stage, that is, a period of struggle during adjustment or adaptation; and third, a stage of exhaustion, that is, a phase of collapse when adaptation is lost due to strong and long-lasting stress [90]. In animals, the GAS can vary among individuals and is strongly influenced by the types and intensities of stressors [89].

The stress response cascade has been classified into three stages: primary response, secondary response, and tertiary response [34,35,38,47,91]. The primary response includes the initial perception of a stressor, which leads to the activation of a neuroendocrine cascade alarming the mobilization of biological systems [34,38,39]. The neuroendocrine response results in the activation of various physiological processes to help the body to adapt to and resist against the stressor. The tertiary response is characterized by the loss of adaptive capacity and an exhaustion of biological systems.

There are distinctions between the primary responses of invertebrates (i.e., crustaceans) and vertebrates (i.e., fish) (Table 2). The distinction between aquatic vertebrates’ and invertebrates’ responses to stress is mainly distinguished by the presence of the vertebrae, hormones, and bioamine products. In crustaceans, the primary response is the rapid and consistent release of crustacean hyperglycemic hormones (CHHs), which have similar effects as cortisol and corticosterone in vertebrates, from the sinus gland in the eyestalks [38,92], which is different from the response in other Arthropods, such as insects. Insects synthesize adepokinetic hormone (AKH) and octopamine (OA) [42] as neurohormones in the brain; these are similar to the neurotransmitter norepinephrine [88]. In vertebrates, such as fish, the primary response involves the secretion and synthesis of corticosteroid hormone (cortisol) and catecholamines (mainly adrenaline and noradrenaline, also known as epinephrine and norepinephrine) in both the hypothalamus and pituitary [34,39].

Invertebrates and vertebrates show relatively similar secondary and tertiary responses upon exposure to stress. Secondary responses to stress include alterations in the cardiovascular system, hydromineral balance, respiratory responses, and immune system function. These responses can affect blood constituent concentrations, such as ions at the cellular level, and the expression of heat shock proteins [33,34,35,36,47,91]. Additionally, secondary responses involve the mobilization of intracellular glycogen and lactate, leading to an increase in hemolymph glucose levels and resulting in metabolic acidosis due to elevated glucose metabolism and lactate accumulation in the bloodstream [38,93]. Tertiary responses encompass changes in behavior, growth, the immune system, and reproduction [34,36,38,47,91].

### 3.1. Primary Response

**Figure 2:**
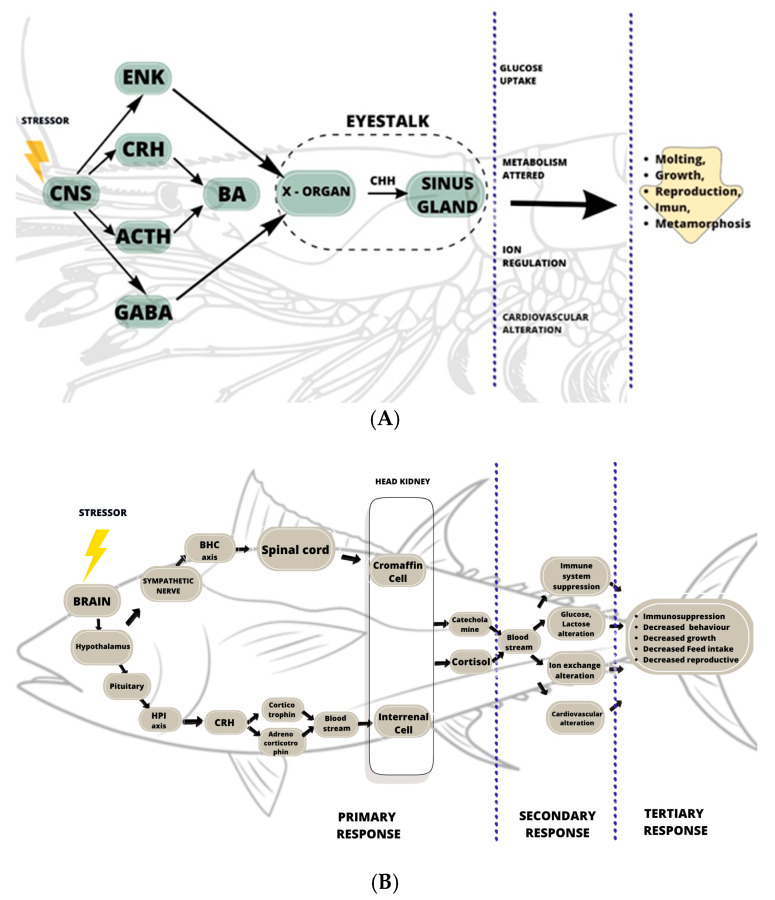
Stress response in crustaceans (**A**) and teleosts (**B**). ENK, enkephalin; CRH, corticotropin-releasing hormone; ACTH, adrenocorticotropin hormone; GABA, ɣ-aminobutyric acid; BA, bioamines (dopamine, octopamine, serotonin/5-HT); CHH, crustacean hyperglycemic hormone; HPI, hypothalamus–pituitary–interrenal; BHC, brain–hypothalamus–chromaffin; CRH, corticotropin-releasing hormone.

The primary response to stress is the initial response that occurs when an animal perceives the stressor and triggers an alert in the body. This alert involves a hormonal release that activates various pathways to maintain homeostasis. Invertebrates and vertebrates differ in their stress response systems (Figure 2).

In vertebrates, the endocrine system includes the hypothalamus, pituitary, and various endocrine glands. Conversely, invertebrates like crustaceans lack these organs and instead have an integrated neuroendocrine system [46]. Crustaceans, such as shrimp, have a central nervous system with a double ventral nerve cord linked by ganglia, primarily located at the anterior end and acting as the brain [88]. When crustaceans perceive stress, their central nervous system (CNS) or ganglia are activated [38,46] and release stress hormones, such as corticotrophin-releasing hormone (CRH) and adrenocorticotropic hormone (ACTH), as well as neurotransmitters like GABA/ɣ-aminobutyric acid and enkephalin [21,46]. Research indicated that crustacean neurotransmitters, including biogenic amines such as catecholamines (epinephrine, norepinephrine, and dopamine) and serotonin (5-HT) [10,21,46,94], also act as second messengers in response to stressors such as escape behavior, locomotion, and aggression [94] and are associated with various immune responses [46]. Biogenic amines are produced in the central nervous system and eyestalk, although the exact production site in the eye remains unidentified [10].

In crustaceans, the primary neuroendocrine organs are the pericardial organs and the X-organ–sinus gland (XO-SG) complex located in the eyestalk [46], with the XO-SG serving as the central neuroendocrine system [95]. These structures are functionally analogous to the hypothalamus–pituitary system in vertebrates and are crucial for producing important hormones for crustaceans [20,38,43]. The XO-SG system produces several hormones, including the crustacean hyperglycemic hormone (CHH), molt-inhibiting hormone (MIH), the vitellogenesis-inhibiting hormone (VIH), also known as the gonad-inhibiting hormone (GIH), and the mandibular organ-inhibiting hormone (MOIH) [95]. The sinus gland nerve connects the X-organ (XO) to the sinus gland (SG), and, when necessary, the SG receives neuropeptide hormones produced by the XO, stores them, and releases them via the axon. This implies that the XO-SG system plays a critical role in regulating various physiological processes, including molting, reproductive maturation, metabolic adaptation, and the immune response, primarily through peptide hormones in response to environmental changes [20,46].

One of the hormones synthesized and secreted by the X-organ–sinus gland (XO-SG) system is the crustacean hyperglycemic hormone (CHH), which is a neuropeptide composed of amino acids. This hormone plays multiple vital roles in crustacean physiology, including the regulation of energy metabolism, maintenance of ionic homeostasis, control of ammonia excretion and metabolism, and mediation of stress responses [23,96,97,98]. The release of CHH from the XO-SG system is regulated by CRH and ACTH, along with biogenic amines [38,42,46,94]. CHH, produced in the medulla terminalis ganglionic organ X and released by sinus glands in the eyestalk, functions similarly to cortisol in fish [38,42]. Despite its relatively straightforward primary response pathway in crustaceans, research on this topic is still limited.

In contrast to crustaceans, vertebrate stress pathways are more complex due to their advanced neuroendocrine system (Figure 2B). Stress response pathways across vertebrates, from fish to humans, exhibit remarkable similarities [33]. These pathways involve catecholaminergic and steroidal stress hormones, which facilitate oxygen uptake and transfer, mobilize energy, redistribute energy away from growth and reproduction, and cause immunosuppression. When the central nervous system (CNS) detects a stressor, it initiates a physiological stress response by activating the neural alarm system, which includes the brain–sympathetic–chromaffin (BSC) axis and the hypothalamus–pituitary–interrenal (HPI) axis [33,99]. The BSC axis activates within seconds [91], rapidly releasing catecholamines into the blood, which complicates studies in vertebrates [34]. The BSC axis influences the cardiorespiratory system by increasing ventilatory and heart rates, cardiac output, and blood flow, with glucose and adrenaline serving as primary mediators in the gills and muscles [39].

Stress signals from the BSC axis travel through the spinal cord and sympathetic ganglia, leading to catecholamine release from chromaffin cells in the head kidney via sympathetic nerve fibers. This process initiates the release of adrenaline (A) and noradrenaline (NA) into the bloodstream. The HPI axis is activated after longer exposure, typically within minutes [91]. Activation of the HPI axis stimulates the release of corticotropin-releasing hormone (CRH) and adrenocorticotropic hormone (ACTH) into the bloodstream. ACTH then prompts the interrenal cells of the head kidney to synthesize and secrete cortisol into the bloodstream [34,100,101,102]. The HPI axis helps reorganize resources by increasing catabolic pathways, supplying glucidic sources, processing fatty acids for energy, and suppressing high-cost energy processes such as immune responses, with plasmatic cortisol levels acting as a major mediator [39].

### 3.2. Secondary Response

The secondary response of stress in both vertebrates and invertebrates involves hormonal changes that circulate through the bloodstream or hemolymph, impacting several signaling pathways and leading to metabolic alterations. This response is characterized by the release of stress hormones, such as cortisol, adrenaline (epinephrine), non-adrenaline (norepinephrine), and crustacean hyperglycemic hormone. These hormones target organs like the hepatopancreas, hemocytes, and muscles, influencing physiological processes [94,102].

In invertebrates, the stress hormone and bioamines increase rapidly, with the timing of cortisol elevation varying with species, life stage, and stressor characteristics [34,36]. Under hypoxia conditions, stress indicators in Kuruma and *L. vannamei* emerge at different times. In Kuruma shrimp, MDA levels spike after 3 h of exposure, while in *L. vannamei*, MDA levels reach their peak around 6 h after exposure, which indicates a slightly delayed oxidative stress response compared to Kuruma shrimp, and these levels generally decline during the reoxygenation phase, reflecting partial recovery from lipid peroxidation damage. These stress indicators increased significantly during prolonged hypoxia, suggesting that the physiological coping capacity of the species may begin to decline beyond acute exposure durations [12,103].

Under ammonia stress, similar patterns emerge, but with different dynamics. In *L. vannamei* exposed to ammonia stress (20 mg/L), biogenic amine levels increased 6 h after exposure, followed by a decrease in the subsequent hour [20]. Meanwhile, in Kuruma shrimp exposed to ammonia stress (42 mg/L), catalase and glutathione S-transferase increased steadily from 6 to 24 h after exposure, before declining at 48 and 96 h [27]. Additionally, a previous study showed that *L. vannamei* exposed to low-pH stress (pH 6.5, near-normal conditions) showed increased enzyme activity (SOD, GPx dan Carboxypeptidase B-like) 48 h post-exposure. However, exposure to high-pH stress (pH 9.7) triggered an earlier increase in hemolymph clottable protein-like, serine proteinase inhibitor B3, and calcium-activated chloride channel regulator 1-like at 24 h [104].

The release of stress hormones induces physiological changes, such as alterations in blood pressure, heart rate [101,102,105], glucose and lactate concentrations [101,102,106], and behavior [34]. These changes reflect the organism’s attempt to reorganize the energy resources [34,101,102,107]. Secondary stress responses also include hematological imbalances, the production of molecular chaperones or heat shock proteins (HSP), and other cellular responses [47]. In fish, cortisol reduces ghrelin production in the stomach and stimulates leptin production in the liver, which can lead to reduced food intake and slower swimming speeds [100,108,109]. In crustaceans, stress reduces the utilization of feed as an energy source for growth, as seen in crayfish [88]. In *L. vannamei*, exposure to heavy metal stress damages the intestinal wall, impairing nutrient absorption [32]. However, the pathway linking stress, gut wall damage, and appetite regulation remains unclear. Overall, metabolic alterations, cardiovascular changes, cellular stress responses, redox regulation, osmoregulatory or hydromineral disturbance, and immune function adjustments are all classified as secondary responses to stress.

#### 3.2.1. Metabolic Alterations

The liver in fish or the hepatopancreas in crustaceans is the primary organ targeted by hormones that enable animals to metabolically adapt to stress. In response to stress, metabolic alteration primarily aims to mobilize immediate energy, mainly in the form of glucose, to restore homeostasis following a stressor. Hyperglycemia is one of the most common metabolic responses upon exposure to stress in many crustacean species [94]. Glucose, the primary energy source, is synthesized in the liver or hepatopancreas through glycogenolysis or gluconeogenesis and is stored as glycogen. During stress or when glycogen reserves are depleted, the body shifts to utilizing lipids and proteins to maintain blood glucose homeostasis. This metabolic adjustment is supported by previous studies showing that stress exposure induces significant changes in gene expression and metabolite profiles associated with glycolysis, gluconeogenesis, fatty acid and amino acid metabolism, and antioxidative pathways [110,111,112]. The regulation of glucose level in the hemolymph is mainly controlled by CHH, which is stimulated by serotonin [113].

Free fatty acids (FAAs) are stored in the body in the form of triacylglycerol (TAG), which can be mobilized when the animal is exposed to stressors, requiring energy to restore homeostasis. However, the exact mechanism of FFA utilization in the form of TAG remains poorly understood [114]. Similarly, the mechanisms related to the utilization of free amino acids (FAAs) for overcoming stress are also unclear [115]. A review by Lee et al. [114] concluded that exposure to different environmental stressors leads to changes in lipid metabolism, which include obesity, changes in fatty acid, TAG, and sterol compositions, and changes in the area of neutral lipids.

Although the molecular interaction of metabolic and energy changes in the crustacean body remains unclear, several studies in *L. vannamei* highlighted energy allocation under stress. Under heat stress, genes involved in ATP synthesis and utilization were downregulated in the hepatopancreas and gills, while protein turnover is almost completely halted. Conversely, genes involved in ATP synthesis and energy-consuming processes are upregulated in muscle, reallocating energy to heat protection and antioxidant system [116]. Similar findings by Nguyen et al. [117] showed an increase in TCA cycle intermediates and a decrease in amino acid and fatty acid metabolism under hypoxia stress, leading to hyperglycemia via a Wargburg-like effect. The process enhances ATP production for stress response through the induction of HSPs.

Furthermore, findings from some studies [62,118] demonstrated that exposure to stressors such as ammonia, nitrite, or hypoxia alters the expression of genes and activities of enzymes related to protein, lipid, and carbohydrate metabolism. For instance, energy-related genes such as peroxisomal acyl-coenzyme A oxidase 1-like were downregulated under salinity, high-pH, and nitrite stress in the hepatopancreas of *L. vannamei*, indicating that multiple stressors impact specific molecular pathways and biological processes [104].

Notably, there were distinct stress responses between the hepatopancreas and muscle: metabolic activity in the muscle increases temporarily, while gene expression in the hepatopancreas tends to decrease more stably. This suggests that the shrimp exhibit differences in energy regulation when adapting to stress, with the hepatopancreas reducing energy expenditure and muscle increasing energy expenditure to protect the body and maintain homeostasis [116,118,119].

#### 3.2.2. Unfolded Protein Mechanism

Stress response at the cellular level involves multiple mechanisms, one of which is unfolded protein (UPR), leading to endoplasmic reticulum (ER) stress. The ER is a large organelle responsible for lipid and steroid synthesis, Ca^2+^ homeostasis and storage, carbohydrate metabolism, and protein synthesis. ER stress causes defects in lipid metabolism, apoptosis, autophagy, reduced respiratory activity, and oxidative phosphorylation. As the cell’s primary Ca^2+^ storage site and the location for protein biosynthesis, folding, and assembly, the ER plays a crucial role in cellular function [120,121].

When stress disrupts cellular balance, eukaryotic cells respond by downregulating protein synthesis, increasing the expression of chaperone-encoding genes and other proteins that prevent polypeptide aggregation and degrading misfolded proteins. This coordinated response is triggered by an intracellular signaling cascade known as the “Unfolded Protein Response” (UPR) [120]. Research on the UPR system in invertebrates is limited compared to vertebrates. However, existing evidence has revealed marked differences in the UPR system between the two groups. Invertebrates possess only one activating transcription factor 6 (ATF6) gene, whereas vertebrates have ATF6⍺ and ATF6β, GADD34 (Growth arrest and DNA-damage-inducible 34), and CHOP (C/EBP-homologous protein). Although many studies have used transcriptional analysis to provide a general framework for understanding shrimp stress mechanisms at the cellular level, certain aspects remain unclear [122].

Several UPR-related genes have been detected in crustaceans in response to different stressors, including Bip (binding immunoglobulin protein), XBP1 (X-box binding protein 1), IRE1 (inositol-requiring transmembrane kinase/endoribonuclease), eIF2α (eukaryotic translation initiation factor α), PERK (PKR-like ER kinase), ATF6, ATF4 (activating transcription factor 4), calnexin, calreticulin, HSP10, HSP21, HSP37, HSP40, HSP60, HSP70, HSC70, HSP90, and PD1 (Protein disulfide isomerase) [123]. The UPR is controlled by three signaling pathways, that is, IRE1, Protein Kinase RNA-like ER kinase (PERK), and ATF6. During ER stress, the upstream non-folding protein recognition factor IRE1 dimerizes and auto-phosphorylates, enhancing endonuclease activity. IRE1 then specifically detects the XBP1 precursor mRNA and excises a particular intron, resulting in the splicing of the XBP1 precursor mRNA. This splicing event combines two open reading frames (ORFs) to encode an active transcription factor: XBP1s [122,123]. XBP1s are translocated to the nucleus and initiate the transcription of downstream genes involved in UPR. These genes encode protein chaperones that promote protein folding, maturation, and trafficking, as well as components of the ER-associated degradation (ERAD) pathway that remove accumulated misfolded proteins in the ER. However, if the chaperones fail to restore homeostasis in the ER, apoptosis is triggered to eliminate the stressed cells [123].

The PERK signaling pathway is implicated in ER stress. PERK activation induces the phosphorylation of eIF2, which globally decreases protein synthesis while promoting the translation of ATF4. ATF4 regulates apoptosis-related genes, such as CHOP, and increases the expression of ER chaperones (HSP) and the oxidative response [120,122,124].

**Figure 3:**
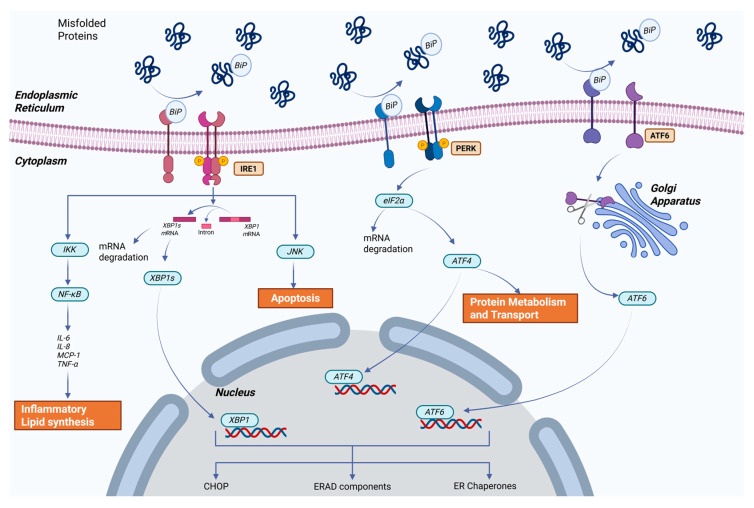
ER stress and UPR signaling pathway.

During ER stress, ATF6 is transported from the ER to Golgi, where it is cleaved by the site-1 protease (S1P) and site-2 protease (S2P). The active ATF6 is translocated to the nucleus and binds to promoters of various genes involved in the UPR, regulating the transcription of downstream effectors such as CHOP, BiP, and XBP1 [120,122]. ER stress also activates the ERAD pathway, the calcium signaling pathway, and additional signaling pathways, including the mitogen-activated protein kinase (MAPK) signaling pathway [122]. Similarly to IRE1 pathway, ATF6 initiates the transcription of HSP and ERAD components [124] (Figure 3).

Heat shock proteins (HSPs) are molecular chaperones that play a crucial role in mitigating stress. Their expression can triple in response to various forms of stress, including physical, chemical, and biological [125]. HSP activation is regulated by Heat Shock Factors (HSFs), which upregulate HSP production to protect cells from damaged or misfolded proteins [110,111,126] by binding to the Heat Shock Element (HSE), which mediates the transcription of heat shock genes [111]. A previous study conducted by Zhou et al. [123] showed that HSF gene expression is highest in the lymphoid organs of stressed *L. vannamei*, with the lowest levels found in the eyestalk. Additionally, stress induces an increase in *HSF* gene expression in *Penaeus monodon* hemocytes [110].

Although the exact mechanism by which HSF influences HSP function remains unclear, studies have suggested that HSF inhibits the NF-κB and Jak/Stat pathways during ammonia stress, leading to a weakened immune response and reduced antiviral activity [126]. Commonly studied HSPs in shrimp stress research include HSP70, HSP90, HSP60, HSP40, and HSP10 [111,126]. Among these, HSP70 showed the most sensitive and tissue-specific gene expression in response to stress, playing a crucial role in protecting cells during adverse conditions [111]. Research on temperature-related stress indicated that HSP70 expression increases significantly in *L. vannamei* and *P. monodon* shrimp when exposed to extreme temperatures of 20 °C and 34 °C, respectively, but stabilizes at near-normal temperatures [69,112]. A previous study [127] found that HSP70 and HSP90 gene expression increased under high-temperature stress but decreased under hypoxic conditions, highlighting how different stressors affect gene regulation associated with stress proteins. Moreover, Niu et al. [72] reported that when the marine copepod *Tigriopus japonicus* was exposed to saxitoxins for 48 h, the HSP70 level increased more than fourfold, indicating cellular stress and disruption of signal transduction pathways. Multi-transcriptomic analysis reveals that HSP gene expression varies by tissue type, with each tissue responding uniquely to environmental stressors [128].

#### 3.2.3. Redox Regulation (Antioxidant)

Various stressors may disrupt the redox state of an organism, including crustaceans, leading to oxidative stress, which is characterized by an imbalance between an excessive production of reactive oxygen species (ROS) and insufficient antioxidant defenses [129,130]. Oxidative stress results in the production of ROS and reactive nitrogen species (RNS), causing damage to DNA and proteins, lipid peroxidation, apoptosis, and overall cell damage [122,130]. Organisms counteract oxidative stress through several signaling pathways and enzymes, including superoxide dismutase (SOD) and glutathione reductase (GR). In crustaceans, various enzymes related to redox reactions have been studied, such as SOD, MnSOD, MDA, GPx, thioredoxin, peroxidase, catalase, NADPH-oxidase, and aldehydeoxidase [122].

The maintenance of the redox environment in vivo relies on the reduced glutathione (GSH)-oxidized glutathione (GSSG) cycle [117,122]. GSH, containing an active sulfhydryl (SH) group, is easily oxidized and dehydrogenated. GSH is reduced to H_2_O by GPx, which then oxidizes to GSSG. GSSG is subsequently reduced to GSH by GR, maintaining the scavenging of free radicals in vivo [122].

There are several molecular signaling processes related to the redox state in aquatic organism. This includes Nrf2 signaling, Notch signaling, PPAR signaling, MAPK signaling, NF-_k_B signaling, and dTLR2-MyD88-NF-_k_B signaling [131]. Since the redox process requires oxygen, the body suppresses the GSH pathway in ROS regulation under hypoxic conditions. A previous study demonstrated that when exposed to hypoxia, Penaeid shrimp downregulates the synthesis of metabolites associated with the glutathione pathway (cysteine, glutathione, glutamic acid, and methionine) [117].

**Figure 4:**
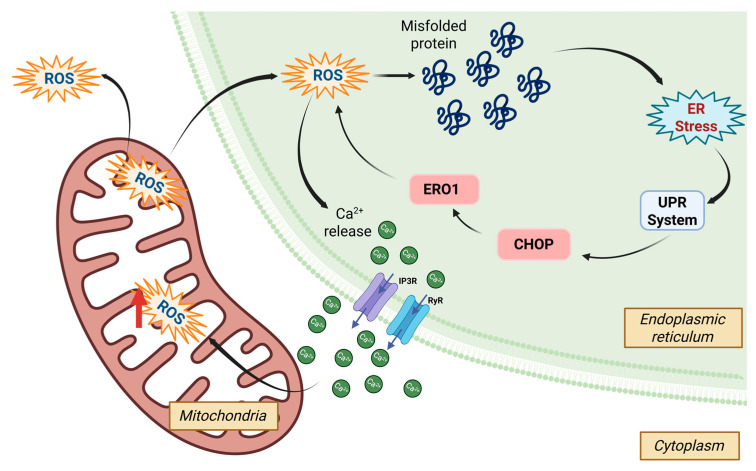
Mechanism of reciprocal relationship between ER stress and oxidative stress in the endoplasmic reticulum and mitochondria.

Oxidative stress and endoplasmic reticulum (ER) stress are strongly connected (Figure 4), although the underlying mechanisms of this interaction remain incompletely understood in crustaceans. In crustaceans, these stress responses are believed to converge on pathways involving calcium signaling, unfolded protein response (UPR), and antioxidant defense systems, which are critical in maintaining cellular homeostasis under environmental and pathogenic stress. While mitochondria are the primary site of ROS generation, the ER contributes approximately 25% to overall ROS production, mainly through the protein-folding process. ER stress activates ROS-sensitive Ca^2+^ channels, such as inositol-1,4,5-triphosphate receptor (IP3R) and ryanodine receptor (RyR), on the ER membrane, leading to the release of Ca^2+^ into the mitochondria. This release further stimulates ROS production. Elevated mitochondrial ROS levels can escape into the ER, exacerbating ER stress. Additionally, components of the UPR contribute directly to oxidative stress; for example, the transcription factor CHOP can increase the expression of ERO1, enhancing the conversion of oxygen into hydrogen peroxide during oxidative folding [121].

#### 3.2.4. Hydromineral Regulation

The secondary response to stress aids in restoring osmotic balance and suppressing the immune system. Upon exposure to a stressor, hydromineral dysfunction occurs due to adrenaline (epinephrine) altering gill blood flow patterns and gill permeability. This alteration favors water movement along its osmotic gradient, either into or out of the body, depending on environmental salinity. Consequently, the role of stress hormones in this context is the restoration of osmotic balance [47,132].

Osmoregulation involves managing ionic fluxes, primarily Na^+^ and Cl^−^ ions, through limiting and compensatory processes such as membrane permeability, epithelial leaks, and active pumping. Maintaining ion gradients is one of the most ATP-intensive activities, making osmoregulation energetically costly. Stress exposure alters the expression of channels or active membrane carriers, including Na^+^/K^+^-ATP_ase_ (NKA) or Na^+^/K^+^/Cl^−^ cotransporters or carbonic anhydrase. Environmental stressors can influence the relationship between osmoregulation and oxidative stress. When the organism experiences stress, it reallocates energy to mitigate stress. Mitochondria, the main energy producers in eukaryotic cells, also generate the majority of ROS and RNS. Elevated levels of ROS and RNS deactivate enzymes involved in osmolyte synthesis and membrane-bound transporters, thus inhibiting osmoregulation [133,134]. In an earlier study, Pinto et al. [28] demonstrated that high ammonia stress not only increased (Na^+^, K^+^)- and V(H^+^)-ATP_ase_ activity but also altered the activity of oxidative stress enzymes (SOD, GST, GR, and G6PDH) and V(H^+^)-ATP_ase_ genes.

#### 3.2.5. Alternation in Immune Responses

Crustaceans exhibit simpler endocrine/neuroendocrine and immune systems compared to vertebrates; however, their stress responses, including the secretion of stress hormones/neurohormones, are comparable to those of vertebrates. During stress exposure, biogenic amine receptors can modulate the immune response by altering second messengers such as adenylyl cyclase (AC), phospholipase C (PLC), cyclic adenosine monophosphate (cAMP), and cyclic guanosine monophosphate (cGMP) [21,135]. Crustaceans primarily rely on a non-specific immune system comprising circulating hemocytes and various active substances released into the hemolymph, including the prophenoloxidase (proPO) system, antibacterial peptides, lectins, and proteinase inhibitors. The neuroendocrine–immunoregulatory network involves physiological and biochemical interactions between the neuroendocrine system and immune system, protecting organisms from stress and diseases [21].

Shrimp have an innate immune system encompassing humoral and cellular responses. The humoral immune response is mainly influenced by the TLR/IMD-NF-κB, JAK-STAT, and RNAi signaling pathways [122]. TLR receptors are activated by pathogen-associated molecular patterns (PAMPs), damage-associated molecular patterns (DAMPs), or homeostasis-altering molecular processes (HAMPs). This activation allows nuclear factor kappa B (NF-κB) to translocate to the nucleus and enhance the transcription of the inflammasome components, pro-IL-1β and pro-IL-18. Concurrently, ROS induce the oligomerization of the NLRP3 inflammasome and activate caspase1, converting pro-IL-1β and pro-IL-18 into IL-1β and IL-18, which promote inflammation and pyroptosis [136].

DAMPs act as an alarm in the innate immune system, promoting pro-inflammatory responses [136]. Besides PAMPs, such as lipopolysaccharide, -1,3-glucan, and peptidoglycan, DAMPs are released upon cell injury caused by biotic and abiotic stresses [137]. There are more than eight types of DAMPs, such as HSPs, high-mobility group protein Box 1 (HMGB1), ATP, uric acid crystals, extracellular DNA, mitochondrial DNA, fibronectin, free fatty acids, and short fragments of free hyaluronan [136]. Peroxiredoxins are also reported as DAMPs [138]. Supplementation with peroxiredoxins can enhance arginine and proline metabolism, suggesting their potential as antibiotics for bacterial diseases in shrimp [137].

### 3.3. Tertiary Response

The tertiary response in both vertebrates and invertebrates occurs when the system becomes dysregulated and fails to return to homeostasis, impairing the metabolic and immune system function [35,47,139]. Although stress hormones have beneficial effects by mobilizing energy during stress exposure, many tertiary responses at the whole-organism level are maladaptive. These include impaired health and disease resistance [140], reproduction [141], growth, learning, and other behaviors such as predator avoidance [38]. In crustaceans, the tertiary response inhibits growth, reproduction, molting, and metamorphosis [38]. For example, when exposed to stressors like high temperature and low pH, crustaceans, particularly *L. vannamei*, experience an increase in metabolic load characterized by the mobilization of glycogen and lipids. This leads to an increase in feed intake without corresponding growth. The metabolic load is further evidenced by a doubling of ammonia excretion and a nearly 50% increase in metabolic oxygen uptake [142]. In both freshwater and seawater decapods, changes in water temperature and salinity do not initially impact growth. However, as the duration and intensity of these stressors increase, survival rates, egg production, and hatching success can significantly decline [48,49,143,144].

## 4. Bioindicators for Stress

**Figure 5:**
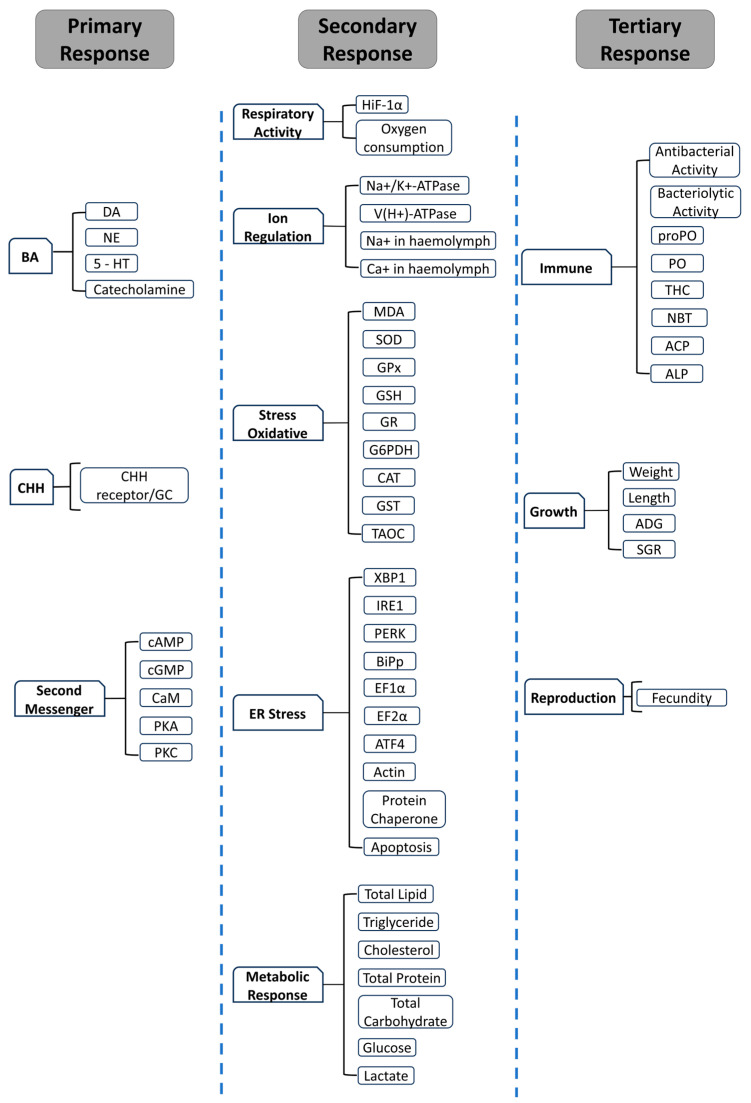
Classification of stress response indicators from primary to tertiary level. Abbreviations: BA: biogenic amine; DA: dopamine; NE: norepinephrine; 5-HT: serotonin; CHH: crustacean hyperglycemic hormone; cAMP: cyclic adenosine monophosphate; cGMP: cyclic guanosine monophosphate; CaM: calmodulin; PKA: protein kinase A; PKC: protein kinase C; HIF-1α: hypoxia-inducible factor 1; MDA: malondialdehyde; SOD: superoxide dismutase; GPx: glutathione peroxidase; GSH: glutathione; G6PDH: glucose-6-phosphate dehydrogenase; CAT: catalase; GST: glutathione s-transferase; TAOC: total antioxidant; ER: endoreticulum stress; XBP1: X-box binding protein 1; IRE1: Inositol-requiring enzyme-1; PERK: RNA-activated protein kinase-like ER kinase; Bip: binding protein; EF1α: eukaryotic initiation factor 1; EF2α: eukaryotic initiation factor 2; ATF4: activating transcription factor 4; FAA: free amino acid; proPO: pro phenol oxidase activity; PO: phenol oxidase activity; THC: total hemocyte count; NBT: nitroblue tetrazolium salt; ACP: acid phosphatase activity; ALP: alkaline phosphatase activity; ADG: average daily growth; SGR: specific growth rate.

Stress significantly impacts not only the metabolism and growth of aquatic animals but also their immunity and disease resistance, making it a crucial factor in disease transmission among aquaculture animals [43]. Early and effective stress mitigation can help the animals immediately return to homeostasis, improve recovery rates, and reduce the risk of chronic stress impact. Therefore, reliable, sensitive, and unbiased stress bioindicators are essential for an early warning system and as parameters to evaluate the efficacy of stress management strategies. To date, the most commonly measured stress biomarkers in crustacea include hormonal, metabolic and cellular, and phenotypic indicators, representing the primary, secondary, and tertiary responses to stress (Figure 5).

### 4.1. Hormonal Indicators

Stress responses in both fish and shrimp are primarily mediated by the neuroendocrine system, involving hormonal and biogenic amine changes. Second messengers facilitate intracellular signaling and activate downstream pathways. In fish, catecholamines and cortisol, produced in the head kidney, are key stress hormones. Conversely, in shrimp or other crustaceans, the crustacean hyperglycemic hormone (CHH), synthesized in the eyestalk, functions analogously to cortisol. Hormonal assays are valuable for detecting stress due to their rapid response to stimuli; however, CHH measurements have traditionally relied on HPLC-based bioassays [98,145], PCR [96,146,147], or ELISA [23,148].

Several studies have utilized hormones as indicators of stress in crustaceans. CHH levels, for instance, increased in response to environmental changes or behavioral stressors, such as predation or migration, signaling stress within the organism. These elevations may occur within 5 [97], 10 [98,145], or even 120 min [149] of exposure to a stressor, depending on its intensity. Furthermore, the amount of CHH secreted varies by crustacean species, type of stressor, and developmental stage. Cortisol levels, commonly used in other taxa, can be influenced by the stress of blood collection itself and are further affected by factors such as diet, season, maturation, and concurrent stressors, complicating their interpretation [150,151].

In addition to its rapid stress response, CHH appears to interact with biogenic amines, adding complexity to its regulatory role. The relationship between CHH and biogenic amines such as serotonin (5-HT), dopamine (DA), norepinephrine (NE), and epinephrine (E) is not fully understood. Some studies indicated a mutually influential relationship. For example, it was demonstrated that 5-HT enhances the release of CHH [148], while silencing the CHH gene leads to a decrease in the levels of DA and other biogenic amines, including NE and 5-HT [96,146,147].

Much evidence showed that alterations in CHH, biogenic amines, and second messengers, such as cAMP, cGMP, calmodulin, protein kinase A (PKA), and protein kinase C (PKC), influence secondary stress response indicators. These include changes in glucose metabolism [147] and also blood glucose [106], ion transport (e.g., Na^+^/K^+^-ATPase activity) [96], and HSP expression [23]. Hormonal indicators are highly sensitive to stress, making them useful for early detection. However, their fast dynamics may lead to challenges in capturing the precise moment of primary stress response, as the organism may have already transitioned to secondary pathways by the time measurements are performed.

### 4.2. Metabolic and Cellular Indicators

After CHH activates second messengers, such as cAMP or Ca^2+^, intracellular signaling cascades are triggered, leading to modifications in various metabolic and cellular pathways. These alterations depend on the type of stressor affecting the organism and can be categorized into five major groups: pathways related to respiratory processes, ion regulation, oxidative stress, ER stress, or energy regulation required to maintain homeostasis.

#### 4.2.1. Respiratory Process

In response to stress, animals experience increased adrenaline, which triggers vasoconstriction and elevates cardiac output. These changes enhance gill diffusing capacity and ion transport, affecting oxygen intake, plasma osmolality, and circulating ion concentrations. Prolonged stress, such as oxygen deprivation, leads to hypoxia, a condition that disrupts the hypoxia-inducible factor (HIF) pathway. This pathway plays a critical role in cellular adaptation to low oxygen levels, primarily through the stabilization and activation of HIFα, a biomarker for hypoxic stress. Interestingly, the presence of HIFα varies across invertebrates. Graham et al. [152] found that among 70 invertebrate species, decapods commonly have the HIFα gene, while only some copepods exhibit the expression of this gene. This suggests that hypoxia adaptation mechanisms differ significantly among invertebrates, depending on their ecological and physiological traits.

In vertebrates, HIFα expression under hypoxic conditions is influenced by the intensity and duration of the stressor [153,154]. Chronic hypoxia resulted in the highest expression of HIFα in the liver, followed by the brain, muscle, heart, and kidneys [153]. In another study, Li et al. [59] demonstrated that hypoxia primarily affects energy metabolism pathways, followed by pathways related to immunity, growth, signal transduction, and hypoxia-specific adaptations. These findings align with other studies showing that hypoxia, or alterations in HIFα, impact glucose metabolism [10,118], glucose transport [154], lactate production [155], oxidative stress [103,118], and the expression of HSPs [127].

#### 4.2.2. Ion Regulation

In both vertebrates and invertebrates, hypoxia-induced stress often disrupts ion homeostasis, emphasizing the critical role of ion-motive ATPase enzymes in maintaining physiological balance under stress conditions. These energetically driven enzymes regulate ion gradients across cell membranes, which are essential in various metabolic pathways. These ions, such as Na^+^, Ca^2+^, and Cl^−^, are abundant in the hemolymph and contribute to critical physiological processes [70]. The regulation of these ions is mediated by ion-motive ATPase enzymes, including Na^+^/K^+^-ATPase, Ca^2+^-ATPase, V-ATPase, and H^+^-ATPase. Furthermore, in both seawater and freshwater fish, cortisol or stress hormones regulate chloride (Cl), potassium (K), and sodium (Na) ions, increasing sodium–potassium adenosine triphosphatase (Na^+^-K^+^-ATPase) activity in the gills, enhancing salinity tolerance [156]. These enzymes utilize energy from ATP to maintain ion homeostasis by driving ion exchange across cell membranes [70,157].

Previous studies demonstrated that both crustacean hyperglycemic hormone (CHH) [148] and cortisol [156], triggered by biogenic amines, increase the activity of ion-motive enzymes such as Na^+^/K^+^-ATPase and H^+^-ATPase through second messengers, including cAMP, PKA, and PKC. This activation enhances ion regulation, such as Na^+^ uptake, in the gills of blue crabs [98]. In addition to affecting Na^+^ uptake, this mechanism alters the concentrations of other ions, including Ca^2+^, Mg^2+^, and K^+^, in the blood of both vertebrates and invertebrates [156,158]. Similar regulatory mechanisms occur in vertebrates, as seen in brown trout and delta smelt, where exposure to environmental stressors like acidification and low salinity influences ion-motive ATPase activity, ensuring ionic balance [159,160].

Ions regulate fluid balance and facilitate the absorption, metabolism, and utilization of many metabolites (fatty acids, amino acids, glucose, monocarboxylate) [157,161]. Na^+^-K^+^-ATP_ase_ and V-H^+^-ATP_ase_ are indicators of transport imbalance, with evidence indicating increased activity in response to stress. For instance, tilapia transferred from freshwater (salinity 0) to seawater (salinity 25 ppt) exhibited significant increases in Na^+^-K^+^-ATP_ase_ activity in the gills and a reduction in glycogen content [162]. Similarly, *L. vannamei* adapted to low salinity showed higher Na^+^-K^+^-ATPase activity compared to the control group [158]. These findings indicate that organisms generate energy as an adaptive response to stressors. Ion concentrations in the hemolymph often serve as physiological indicators, where deviations may signify stress and disruption of homeostatic mechanisms. However, the mechanisms underlying ion regulation in crustaceans remain poorly understood, particularly in response to environmental stressors. Despite the crucial role of ion balance in survival and aquaculture performance, recent studies in this topic are scarce, leaving significant gaps in our understanding of crustacean homeostasis and stress resilience.

#### 4.2.3. Cellular Stress

Alterations in homeostasis affect cellular processes, leading to oxidative and endoplasmic reticulum stress. Stress triggers the production of ROS, which are counteracted by antioxidants from the body or diet. An imbalance between ROS and antioxidants results in oxidative stress, disrupting cellular function [130,133].

The activity of antioxidant enzymes, such as alkaline phosphatase (AKP), acid phosphatase (ACP), total antioxidant capacity (T-AOC), glutathione S-transferase (GST), glutathione peroxidase (GPX), glutathione (GSH), malondialdehyde (MDA), and protein carbonyl (PC), is used as an indicator of oxidative stress [29,130,134]. Their reliability, however, varies with developmental stage and diet, which influence antioxidant efficiency [130]. Studies have shown that in *L. vannamei*, antioxidant enzyme activity can differ between tissues, such as the hepatopancreas and gills, with some authors reporting asynchronous spikes, while others observe simultaneous responses [11,103]. These variations highlight the complexity of antioxidant regulation under stress.

When antioxidant defenses fail to neutralize the excess ROS generated by stress, cellular imbalance intensifies, leading to ER stress. In response, the ER activates the unfolded protein response (UPR), a signaling pathway designed to alleviate the accumulation of misfolded proteins. This process involves intricate intracellular mechanisms that help restore cellular homeostasis [120,121,163]. However, the precise relationship between oxidative stress and ER stress, particularly in crustaceans, remains poorly understood. Recent studies have begun to explore this connection by identifying genes involved in the UPR and ER stress, offering insights into the underlying cellular mechanisms [104,164,165].

The most commonly used indicators of ER stress are Damage-Associated Molecular Patterns (DAMPs), such as HSPs. Among these, HSP70 is the most frequently used, due to its abundance and conservation across species. Other key HSPs, including HSP40, HSP90, HSP60, and Heat Shock Factor 1 (HSF-1), are also utilized as indicators of cellular stress [17,166,167,168,169]. He et al. [138] identified peroxidoxin-1 as a novel DAMP involved in acute liver injury (ALI) in mice. In ALI models induced by acetaminophen (APAP) and carbon tetrachloride (CCl_4_), serum Prdx1 levels increased progressively, correlating with macrophage infiltration and elevated expression of IL-1β, IL-6, and TNF-α. Prdx1-deficient (Prdx1^−^/^−^) mice exhibited reduced inflammation and liver damage, while reintroducing recombinant Prdx1 restored these effects, confirming its role in ALI pathogenesis via NF-κB activation and NLRP3 inflammasome signaling. Despite extensive research on DAMPs in vertebrates, their role in invertebrate stress physiology, particularly in crustaceans, remains largely unexplored.

#### 4.2.4. Metabolic Indicators

Plasma metabolites such as glucose and lactate are frequently used to assess stress conditions in aquatic organisms. Cortisol modulates glucose production through processes like gluconeogenesis and glycogenolysis, while lactate is generated from glucose via anaerobic glycolysis to meet the increased cellular energy demands. Despite their utility, glucose measurements often exhibit variability and are less reliable than cortisol for stress assessment, thus serving primarily as supplementary indicators [150,170]. In addition to glucose and lactate levels in the blood, other metabolic indicators used in previous studies include total lipids, total proteins, triglyceride [79], blood cholesterol, free fatty acids (FFAs) [171], and free amino acids (FAAs) [115]. However, a meta-analysis study by Conneley et al. [172] showed that lactate concentration is a particularly accurate stress marker, although it may be less suitable for chronic stress assessment [172].

Hyperglycemia, characterized by elevated blood glucose and lactate levels, occurs in response to stress and affects blood components such as leukocytes, erythrocytes, hemoglobin, and hematocrit values in fish [151]. Blood profiling is essential for assessing the overall health status of an animal. In crustaceans, similar alterations occur, impacting the total hemocyte count (THC) and differential hemocyte count (DHC) [94]. Consequently, these enzymes are frequently used as biomarkers to assess the degree of stress in fish.

In shrimp, stress-induced cellular damage is often reflected by changes in the activities of enzymes in hemolymph, such as alanine aminotransferase (ALT), aspartate aminotransferase (AST), phenoloxidase (PO), lysozyme (LZM), lactate dehydrogenase (LDH), and creatine kinase (CK). These enzymes are released into the hemolymph following cellular injury or death, and their elevated levels can indicate stress, especially when tissue damage is minimal [135]. Consequently, these enzymes are frequently used as biomarkers to assess the degree of stress in shrimps.

Specifically, ALT and AST are aminotransferases linked to tissue and cellular damage. For example, a study by Li et al. [173] demonstrated that in *L. vannamei*, reduced salinity led to decreased ALT and AST activity, while PO activity increased, indicating damage to hepatopancreas cells. Elevated levels of these enzymes often correlate with cellular damage, oxidative stress, and tissue inflammation, making them useful indicators of shrimp health and well-being. Monitoring the levels of these key enzymes in shrimp populations can thus serve as an effective tool for assessing environmental stress and optimizing aquaculture management practices. However, further research is necessary to better understand the specific mechanisms behind their release and to establish standardized thresholds for their use as reliable biomarkers in different stages of shrimp cultured under different environmental conditions.

### 4.3. Phenotypic Indicators

Stress significantly affects the performance of both fish and crustaceans, including growth, disease resistance, and metabolism [108]. Although easy to measure, these phenotypic traits or physiological indicators have limited specificity. Key indicators of welfare include growth and fitness, condition factor (length–weight relationship), feed consumption, metabolic rate, and behavioral changes, such as aggressiveness, swimming performance (speed, intensity, and duration), gill ventilation rate, color changes, predator–prey dynamics, malformations, and pathological conditions. Various organosomatic indicators also reflect general health [151].

In certain situations, traditional stress biomarkers are difficult to interpret and may lead to challenging diagnoses. Therefore, identifying and comprehending alternative tools is crucial for aquatic organism welfare [35]. Recent studies have combined traditional stress biomarkers with genomic tools to understand the entire process of stress responses in both acute and chronic stages [47] and to identify specific and early indicators of stressors [35]. In aquaculture, technologies such as genomics, transcriptomics, proteomics, and metabolomics have been employed to enhance knowledge and understanding of safety, production, quality, and health. The application of various omics approaches—genomics (the study of DNA variation), transcriptomics (the analysis of genome characterization by gene expression, mRNA), proteomics (the study of cell and tissue protein expression), and metabolomics (the study of chemical processes involving small molecules known as metabolites)—is necessary for aquaculture research. By integrating multiple omics platforms, it is possible to comprehend the complexity of biological interactions and understand how cells and organisms adapt, function, grow, and develop.

## 5. Stress Mitigation Strategy

Stress mitigation strategies for the aquaculture industry may be implemented following two approaches: first, by system modification to reduce the magnitude of stress, and second, by improving the robustness of the cultured organisms to stress. System modification includes efforts to reduce culture intensity (density per unit area) [174], maintaining good water quality [175], and improving biosecurity [176], as well as reducing the impact of climate on the culture by performing the culture in a closed indoor system [177]. The second approach includes genetic improvement and dietary interventions to improve the animals’ robustness against stress.

Selective breeding programs targeting genetic traits that are related to stress robustness have been developed to maintain a sustainable aquaculture production [178]. In crustaceans, such programs are primarily applied to shrimp, with a strong emphasis on enhancing disease resistance [179], which remains a major challenge in shrimp farming. However, the implementation of selective breeding is often limited for small- and medium-scale farmers, as it requires substantial financial investment, long-term commitment, and access to sophisticated breeding facilities.

This limitation becomes even more pronounced when considering advanced genomic and omics-based approaches for stress mitigation, which are currently not feasible for direct application in most farm-level operations. Nevertheless, these approaches play a crucial role in identifying molecular biomarkers associated with stress responses. Such biomarkers can serve as early indicators of stress exposure and allow for rapid diagnostics, thereby supporting more timely and precise farm management interventions.

Several studies have investigated the heritability of stress tolerance traits and their underlying molecular mechanisms. For example, Anastasiadi et al. [180] demonstrated that DNA methylation patterns in European seabass can be influenced by early-life thermal exposure, potentially affecting the stress phenotype of the offspring. Similarly, Fellous et al. [181] found that a 2 °C temperature increase altered the expression of DNA methylation-related genes in seabass larvae. In marine sticklebacks, adaptation to elevated temperatures induced dynamic, temperature-sensitive methylation changes throughout development [182]. However, similar studies in shrimp and other crustaceans are still lacking, pointing to a significant gap in our understanding of how stress resilience may be inherited in these taxa.

Apart from providing balanced and optimum nutrients for normal growth through diet, some research demonstrated that stress mitigation can be achieved through the addition of some specific nutritive compounds, like amino acids, vitamins, and minerals or non-nutritive compounds such as probiotics and organic acids [47]. Tryptophan, for instance, has been considered to be a promising stress mitigator in fish. This amino acid is a precursor of serotonin (5-hydroxytryptamine, 5HT) and melatonin, which play an important role in stress-coping ability and behavior in several fish and crustacea [183]. Increased dietary tryptophan leads to an increased availability of this amino acid for uptake into the brain, where it can later be converted to 5-HT with the facilitation of tryptophan hydroxylase and aromatic L-amino acid decarboxylase in the presence of vitamin B6 [184]. Dietary tryptophan has been shown to reduce the levels of stress indicators in some fish species, such as *Channa punctatus* [185], *Lates calcarifer* [186], *Oreochromis niloticus* [187], *Argyrosomus regius* [188], *Chirrhinus mrigala* [189], and brown trout [190]. On the other hand, the role of tryptophan in mitigating stress in crustaceans remains underexplored.

While extensive studies have demonstrated tryptophan’s modulatory effects on serotonergic activity, stress responses, immune regulation, and antioxidant capacity in various teleost species [191], similar investigations in crustaceans are scarce. This highlights a critical research gap and the need for species-specific studies to better understand the physiological pathways involved. Nonetheless, emerging studies provide promising evidence. For instance, in crayfish, anxiety-like behavior is influenced by the intensity of stress stimuli, with levels of anxiety increasing as brain 5-HT concentrations rise [192]. In *L. vannamei*, dietary tryptophan supplementation at 0.36% has been shown to significantly elevate 5-HT levels in the hepatopancreas, eyestalk, and hemolymph, suggesting enhanced neurochemical resilience to stress [193]. Additionally, Jin et al. [194] demonstrated that dietary tryptophan at 3.61 g/kg optimized growth performance and feed conversion efficiency in *L. vannamei* reared under low-salinity conditions, while also improving protein efficiency, reducing stress-related enzymes (AST and ALT), and enhancing metabolic profiles, including hemolymph amino acid concentrations. In juvenile mud crabs, tryptophan supplementation at 0.75–1% reduced aggressive behavior and improved survival, correlating with increased 5-HT levels after social conflict [195]. These findings underscore the potential of tryptophan as a dietary intervention for stress mitigation in crustaceans, warranting further investigation into species-specific dose responses and underlying molecular pathways.

## 6. Future Directions

Environmental stressors are currently evaluated by measuring their effects on various biological indicators, including population density, growth, reproduction, behavior, physiology, and immune responses. Environmental stress may trigger some sublethal effects, such as mutations and epigenetic changes, that can be transgenerationally inherited, influencing phenotypic plasticity and increasing vulnerability to diseases, tissue pathologies, and changes in social behavior and biological invasions [196]. In the future, stress monitoring should be performed in real time with high precision, allowing for early mitigation and thus reducing the risk of loss. However, compared to terrestrial animal husbandry, research and technological advances in stress mitigation in aquaculture, especially crustaceans, are relatively far behind.

Internet of things (IoT)-based technology has been successfully implemented in cattle production to monitor stress levels. In aquaculture, real-time stress monitoring in fish has advanced using biosensors that track biomarkers such as blood glucose [197,198] or visualized behavior [199,200]. This advancement may not be applicable to crustacean aquaculture yet. There remains insufficient knowledge of crustaceans’ stress responses, which limits the development of biosensors. Previous research has mainly focused on secondary physiological responses, such as changes in intestinal tissue structure and appetite [201] and energy allocation [13,22,116,117,202]. However, little attention has been given to the molecular alterations that drive these changes. The development of multi-omics tools presents an opportunity for a comprehensive understanding of the responses of aquatic animals to stressors at both primary (molecular and cellular) and secondary (physiological and behavioral) levels. However, several critical gaps remain unaddressed. First, there is a lack of integrated studies linking molecular, physiological, and behavioral stress responses, particularly in crustaceans. Second, while biosensors have proven effective in fish, their application in crustaceans is hindered by the absence of validated, quantifiable biomarkers. Third, the long-term and transgenerational impacts of sublethal stressors—such as epigenetic modifications—are still poorly understood.

Future research should prioritize the identification and validation of crustacean-specific stress genes and pathways, the development of non-invasive biomonitoring techniques, and the application of real-time stress tracking technologies tailored to aquatic invertebrates. Exploring these gaps will be essential to advance precision aquaculture and enhance resilience against environmental challenges.

In addition, feed supplementation to mitigate stress still has a lot of room for exploration; examples include the use of alternative ingredients like quinoa husk [203], galacto oligosaccharides (GOSs) [204], or essential oils in feed [205]. In particular, the use of essential oils remains underexplored, with limited studies elucidating their mechanisms of action, especially their antioxidant capacity and role in stress mitigation, highlighting a promising avenue for future research in aquatic animal nutrition.

## 7. Conclusions

In essence, stressors in aquaculture are inevitable and can have a significant impact on sustainable production. Thus, it is vital to explore effective strategies for mitigating stress in cultured species. A comprehensive understanding of the stress responses in these species, particularly crustaceans, is crucial before implementing any interventions, whether through changes in rearing practices, technological monitoring, or dietary modification.

This review highlights the major physiological pathways involved in stress responses in crustaceans and emphasizes their relevance to aquaculture. It also identifies critical knowledge gaps—such as the limited integration of molecular, physiological, and behavioral data; the absence of validated, crustacean-specific stress biomarkers; and the lack of tools for real-time, non-invasive monitoring. Furthermore, the long-term and transgenerational effects of sublethal stressors remain poorly understood.

Addressing these gaps through multi-omics approaches, advanced sensor technologies, and tailored nutritional strategies will be essential to developing precise, adaptive stress management frameworks. Encouraging research in these directions is key to enhancing crustacean resilience and ensuring the sustainability of aquaculture under accelerating environmental change.

## Figures and Tables

**Table 2:** Pathway differences between aquatic invertebrates and vertebrates.

	Invertebrate Crustacean	Vertebrate Fish
Signaling system	Neuroendocrine system	Endocrine system
Axis	XO-SG axis	BSC axis and HPI axis
Hormones	Crustacean hyperglycemic hormone/CHH	Cortisol and Catecholamine
Biogenic amines	Norepinephrine, GABA, dopamine, serotonin	Norepinephrine, GABA, dopamine, serotonin
Major signaling pathway	Central nervous system	Hypothalamus, spinal cord, and pituitary
Stress hormone-producing organ	Eye stalk	Head kidney

## Data Availability

The data underlying this study’s findings are accessible from the corresponding author upon a reasonable request.
